# Supplementary material for: Prescriber Commitment Posters to Increase Prudent Antibiotic Prescribing in English General Practice: A Cluster Randomized Controlled Trial
Source: Antibiotics (Basel). 2020 Aug 7;9(8):490. doi: 10.3390/antibiotics9080490 (PMC7569839; doi:10.3390/antibiotics9080490)
Supplement: Supplementary file 1 [file antibiotics-09-00490-s001.zip › Additonal File 3. Intervention description BCT-T V1.docx]

**Additional File 3. Intervention description according to the Behavior Change Technique Taxonomy (BCT-T) V1**

| **Commitment poster**  **The primary target for the commitment poster is prescribers. Only prescriber directed BCTs are coded.** | **BCT** | **BCT description** |
| --- | --- | --- |
| **Individual commitment**  Active process of reading, signing and adding photo to poster and the commitment banner itself – stating: ‘I am committed to safe antibiotic prescribing to protect our patients’ health.’  **Practice commitment**  We are committed to combating drug resistant infections to protect your health and well-being. We will only prescribe antibiotics when it is the appropriate and necessary treatment. We do not prescribe antibiotics for colds and other self-limiting infections. | 1.9 Commitment | 1.9 Ask the person to affirm or reaffirm statements indicating commitment to change the behavior Note: if defined in terms of the behavior to be achieved also code 1.1, Goal setting (behavior) |
| **AMS message from GP to patient**  We want to give you some information about antibiotics and caring for your health. Antibiotics help us to fight serious infections caused by bacteria such as meningitis, kidney infections and MRSA. But antibiotics can also cause side effects like skin rashes, diarrhoea, stomach pain, thrush or vomiting. If your symptoms are from a virus not from bacteria, like most colds, coughs, flu and some mild infections, an antibiotic won’t help you get better and you could still get these side effects. Taking antibiotics for illnesses which will not benefit from them encourages bacteria to become resistant to the treatment causing drug-resistant infections. This means that antibiotics are losing their effectiveness. If you take antibiotics unnecessarily this could limit the treatment options available should you have more serious bacterial infections in the future. Antibiotics might not work when you and your family really need them. How can you help? Go to your pharmacist for advice if you have symptoms of the flu, a cold, cough, sinusitis, sore throat or a mild water, skin, eye or ear infections. The pharmacist will give you advice on how to care for your infection and tell you whether you should see your GP. As doctors we will always treat your illness in the best way possible. We will avoid prescribing antibiotics when they could do more harm than good. | 5.1 Information about health consequences  5.2 Salience of consequences  5.3 Information about social and environmental consequences | 5.1 Provide information (e.g. written, verbal, visual) about health consequences of performing the behavior  5.2 Use methods specifically designed to emphasise the consequences of performing the behaviour with the aim of making them more memorable (goes beyond informing about consequences).  5.3 Provide information (e.g. written, verbal, visual) about social and environmental consequences of performing the behaviour |
| Antibiotic prescribing – proceed with caution! Poor prescribing puts patients at increased risk of drug resistant infections. | 5.1 Information about health consequences | 5.1 Provide information (e.g. written, verbal, visual) about health consequences of performing the behavior |
| PLEDGE acronym | 4.1 Instruction on how to perform the behaviour  5.2 Salience of consequences | 4.1 Advise or agree on how to perform the  behaviour  5.2 Use methods specifically designed to emphasise the consequences of performing the behaviour with the aim of making them more memorable (goes beyond informing about consequences). |
| Poster | 7.1 Prompts/cues | 7.1 Introduce or define environmental or social stimulus with the purpose of prompting or cueing the behavior. The prompt or cue would normally occur at the time or place of performance |
| **Automated message**  **The primary target for the automated message is patients and only patient directed BCTs are coded. We expect the intervention to impact upon antibiotic prescribing through fewer patient consultations for self-limiting RTIs.** | 12.1 Restructuring the physical environment | 12.1 Change, or advise to change the physical environment in order to facilitate performance of the wanted behavior or create barriers to the unwanted behavior |
| *“GPs in this practice do not prescribe antibiotics for infections which usually get better on their own such as colds and flu.* ***Please visit your pharmacist for advice.”*** | 3.2 Social support (practical)  8.2 Behaviour substitution | 3.2 Advise on, arrange, or provide practical help (e.g. from friends, relatives, colleagues, ‘buddies’ or staff) for performance of the behaviour  8.2 Prompt substitution of the unwanted behavior with a wanted or neutral behavior |
| ***“GPs in this practice do not prescribe antibiotics for infections which usually get better on their own such as colds and flu.*** *Please visit your pharmacist for advice.”* | 4.1 Instruction on how to perform the behavior  6.3 Information about others approval  9.1 Credible source | 4.1 Advise or agree on how to perform the behavior  6.3 Provide information about what other people think about the behavior. The information clarifies whether others will like, approve or disapprove of what the person is doing or will do.  9.1 Present verbal or visual communication from a credible source in favour of or against the behaviour |
