# Supplementary figures and images for: Prescriber Commitment Posters to Increase Prudent Antibiotic Prescribing in English General Practice: A Cluster Randomized Controlled Trial
Source: Antibiotics (Basel). 2020 Aug 7;9(8):490. doi: 10.3390/antibiotics9080490 (PMC7569839; doi:10.3390/antibiotics9080490)

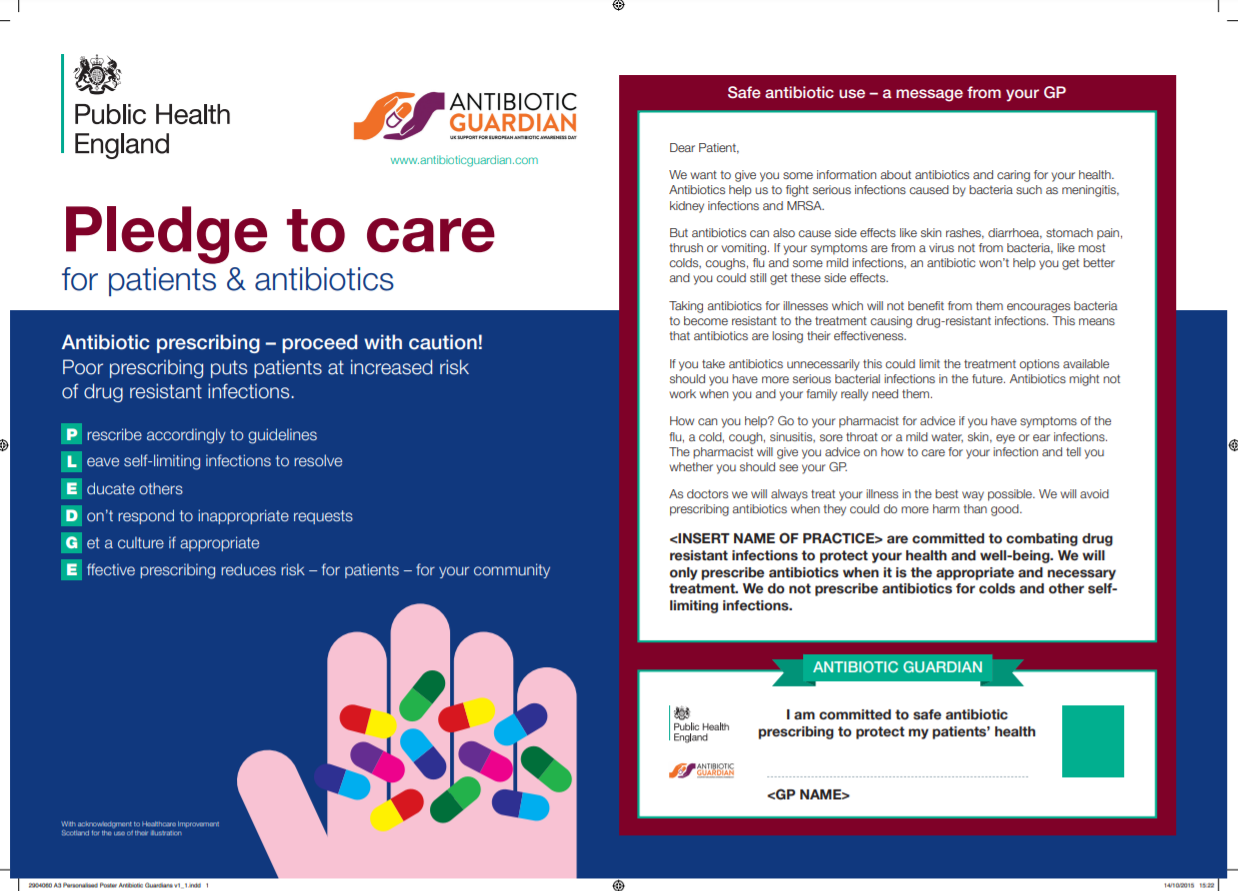

Supplement: Supplementary file 1 [file antibiotics-09-00490-s001.zip › Additional File 2. Commitment poster.docx]
