# Supplementary material for: Prescriber Commitment Posters to Increase Prudent Antibiotic Prescribing in English General Practice: A Cluster Randomized Controlled Trial
Source: Antibiotics (Basel). 2020 Aug 7;9(8):490. doi: 10.3390/antibiotics9080490 (PMC7569839; doi:10.3390/antibiotics9080490)
Supplement: Supplementary file 1 [file antibiotics-09-00490-s001.zip › Additional File 1. Trial protocol.doc]

##### Additional File 1: Trial Protocol

**A Practice Level Intervention to Reduce Antibiotic Prescribing for Self-Limiting Infections in Primary Care**

Public Health England (PHE)

Behavioural Insights Team

6^th^ May 2016, Version 7, amendment 4.

**Contents**

| Context | 3 |
| --- | --- |
| Research Aims | 3 |
| Summary of Interventions | 3 |
| Study Methodology | 4 |
| Recruitment | 4 |
| Inclusion and Exclusion Criteria | 4 |
| Timing | 5 |
| The Interventions | 5 |
| Intervention Fidelity | 8 |
| Outcome Measures | 8 |
| Additional Data | 8 |
| Data Collection | 8 |
| Sample Size | 8 |
| Analysis | 8 |
| Quality Assurance | 9 |
| Roles and Responsibilities | 9 |
| Confidentiality and Ethical Approval | 9 |
| Funding | 9 |
| References | 9 |

**Context**

Tackling antimicrobial resistance (AMR) is one of Public Health England’s (PHE) strategic priorities with the aim to reduce the number of serious infections that are resistant to treatment^[1]^. Inappropriate use and overuse of antimicrobials such as antibiotics is a major driver of antimicrobial resistance^[2]^. The number of antibiotics prescribed in England increased by 6% between 2010 and 2013 and the number of bloodstream infections caused by resistant organisms has also increased over this period^[3]^.

A comprehensive review of over 150 scientific articles by PHE identified key behaviours in support of antibiotic stewardship, and drivers of these behaviours that are amenable to change^[4]^. Evidence in support of previous interventions (such as practitioner education and training or back-up prescribing) is mixed, opening a research opportunity for novel interventions. Results of a behavioural analysis looking at antibiotic prescribing behaviours suggests that new interventions have the potential to mitigate antibiotic resistance and are applicable to the primary care settingError: Reference source not found. General practitioners (GPs) are often the first port-of-call for patients seeking medical help and approximately four-fifths of antibiotics are prescribed in primary care, making this a suitable target for an interventionError: Reference source not found.

Perceived patient pressure and expectation for antibiotic treatment are commonly cited as reasons for doctors prescribing antibiotics ‘unnecessarily^[5]^’. An intervention occurring before an inappropriate appointment or request is made would prevent the need for GPs to refuse antibiotics. Where GPs are in a position to refuse antibiotic treatment during a consultation they need to be committed and supported to do so.

PHE’s Behavioural Insights Team is working with prescribing experts and colleagues from The Department of Health Expert Advisory Committee on AMR & HCAI (ARHAI) to apply behavioural insights to tackle the problem of overuse of antibiotics.

This proposal describes two interventions aimed at reducing patient demand, delivered through a single study in the context of GP practices.

**Research aims**

1. Reduce inappropriate antibiotic prescribing by GPs in primary care in England. Inappropriate prescriptions are those which do not improve patient health outcomes and/or are those which are not recommended by clinical guidelines.

2. Explore clinician and practice manager views of implementing the interventions delivered in the trial and clinicians views on how these influenced their prescribing.

**Summary of interventions**

This project will test the effectiveness, and assess the implementation, of 2 interventions to reduce antibiotic prescribing in GP practices in England. In brief the interventions are:

1) Introduction of a brief recorded message on the GP surgery answer phone explaining to patients that GPs in this practice are committed to prescribing antibiotics prudently and only when the benefits outweigh the risks to the patient’s health, in accordance with national guidelines; and directing patients to their pharmacy for self-care advice for infections that do not usually require antibiotics such as upper RTIs. This aims to address patient demand for appointments and ultimately reduce access to antibiotics by facilitating an expectation that inappropriate antibiotics will not be prescribed.
2) Display a ‘commitment poster’ in GP consulting rooms with a photo of the GP and a signed statement of their commitment to appropriate prescribing of antibiotics. This aims to provide a tool to support GPs, during the consultation, in their decision not prescribe antibiotics where it is not clinically appropriate and also to strengthen the GPs own commitment to appropriate prescribing through this public commitment.

**Study methodology**

The study will be conducted as a non-blinded cluster randomised controlled trial with GP practices as the unit of randomisation. We propose a 3 armed factorial trial to test:

- Intervention arm 1: GP commitment poster alone
- Intervention arm 2: Answer phone and poster
- Control arm

The interventions would be delivered at GP and practice level but the unit of analysis is the practice/month level of prescribing weighted by population and STARPU. Practices would be randomly assigned to receive one of the intervention arms or the control, stratified by CCG. Balance checks will be conducted looking at previous rates of prescribing.

Following the end of the trial, a qualitative study will be carried out interviewing general practitioners about their experiences of following and implementing the interventions in practice and how this influenced their antibiotic prescribing. A short questionnaire will also be sent to practice managers to capture their views of implementing the interventions.

**Recruitment and treatment allocation**

All GPs at a participating practice would need to opt in to the trial.

GPs (and GP practices) will be recruited purposively via Clinical Commissioning Groups (CCG) and one Commissioning Support Unit (CSU). Areas currently involved are CSU North East, NHS CCG Kernow, Liverpool CCG, High Weald, Lewes and Haves and NHS CCG Bedfordshire. A range of other CCGS are now additionally taking part.

The sample will be randomised to the interventions, stratified at the CCG level using an even-split-within-strata method, which ensures numerical balance within each CCG. After randomisation, the sample will be checked for between-conditions balance on patient list size and on prior prescribing rates. In the event that the sample is unbalanced on these variables, the process of randomisation will be repeated.

The research will be positioned as interventions to help address patient demand for inappropriate antibiotic prescribing, recognising that many GPs feel that the ‘blame’ for this increased use of antibiotics is often, and not always fairly, levelled at them, failing to take into account patient demand^6^

Those who opt in will be contacted with further study details and confirmation will be requested of support from all GPs in the practice through return of signed study materials (poster arm) or agreement over email (control, call waiting arms).

**Inclusion and exclusion criteria**

- All GP surgeries within a CCG will be eligible regardless of prescribing rates^[6]^
- Selected GP practices must have an automated call answering system in place which could be adapted to include a short voice message about the practices antibiotic prescribing policy and symptoms for which self-help and pharmacy care might be more appropriate; but which are not already employing such a message
- Practices would not be excluded if they are concurrently implementing alternative AMR interventions and policies as it is expected they may be doing so over the peak flu and prescribing season however data will be captured about other ongoing or recently implemented interventions for AMR
- Practices would be excluded if they were currently implementing an answer phone or commitment poster AMR intervention
- Walk-in centres are excluded.
- Practices which utilise online appointment booking will be excluded if they take more than 20% of bookings online.

**Timing**

The trial will run for a 4 month period during peak flu season between January 2016 and April 2016 due to higher incidence of Acute Respiratory Illness (ARI).

The qualitative study and short survey will commence at the end of the trial after all practices have completed their participation.

**The interventions**

**Intervention 1: Answer Phone Message**

Participating GP surgeries will be those who already have a recorded message in place which is played when a caller rings the surgery during opening hours. Many clinic answer phone messages either inform callers of surgery news (e.g. you can now book appointments online, please book a flu jab) or direct callers who are calling for test results, or emergencies to different options and those seeking an appointment to a separate phone option. Dependent upon local arrangement the message would be played to all callers during opening hours only. After the message has been played, the call will go into the queue to be answered by the receptionist as usual. Surgeries where more than 20% of bookings are taken online will be excluded from the study.

*Intervention rationale*

The call waiting procedure already takes place in most UK GP practices^[7]^, making this a simple and inexpensive intervention to implement. Qualitative market research on antibiotic use in primary care involving interviews with GPs and mothers obtained mixed feedback on the call waiting message conceptError: Reference source not found. A number of GPs supported the idea that call waiting could convey key messages and help manage patient expectations prior to appointments. Similarly some mothers were receptive to this idea, suggesting that it could be useful in allowing them to make an informed decision about whether or not to make a GP appointment. A comprehensive literature review by PHE proposed a series of potential interventions based on the COM-B frameworkError: Reference source not found. Call waiting was suggested as a means of averting unnecessary presentation at source (prior to appointment booking). It is also likely to prime the patient for the authority of the doctor to make the decision on whether or not it is necessary to prescribe antibioticsError: Reference source not found. The proposed answer phone intervention has been created based on language taken from NICE clinical practice guidelines^[8]^, NHS Choices recommendations^[9]^ and existing materials given to patients (such as the RCGP TARGET patient leaflet^[10]^). The message is short in duration (10-12 seconds in length) to reduce hang-ups.

*Logistics*

The answer phone message script will be sent to practice managers of participating surgeries via email. Practice managers will then be responsible for recording and updating their surgery answerphone system.

The proposed message is as follows:

***“GPs in this practice do not prescribe antibiotics for infections which usually get better on their own such as colds and flu.  Please visit your pharmacist for advice.”***

**Practices may add the following to the message if they wish:**

***If you are over 65, making an appointment for a child under 5, have an underlying condition or would still like to see a doctor please stay on the line/press X for an appointment.***

**Intervention 2: Commitment Poster**

A poster will be designed to include a signed statement of commitment to preserving antibiotics. The GP commitment posters will be displayed in consultation rooms and include the relevant GP’s photograph, signature, commitment letter and pledge statement (see below).

*Intervention rationale*

CCGs in Leeds and one health board in Wales have reported implementing a similar “commitment poster” intervention with overwhelmingly positive feedback from clinicians, demonstrating the feasibility and acceptability of this sort of intervention. Across these areas the intervention has been well received, with acceptance in 112 practices across the 3 CCGs in Leeds. However evaluation of these existing self-created interventions is limited. Therefore a similar poster approach is logical, crucially with a usual care control arm set up to evaluate the effectiveness of posters as an intervention. The content for intervention 2 comes from adapted examples of poster copy taken from Meeker et al. (2014)^[11]^ and the CCG initiatives mentioned above. The NICE guidelinesError: Reference source not found, NHS Choices adviceError: Reference source not found and information taken from existing social marketing materials (namely RCGP patient leafletError: Reference source not found) have been incorporated in the poster text to validate clinical content for example, recommendations on which illnesses antibiotics can and cannot clinically treat. The current poster intervention aims to provide a tool to support GPs during the consultation and also to strengthen GP commitment to appropriate prescribing through this public commitment.

*Logistics*

The commitment poster template will be sent to participating surgeries via email. GPs, NMPs and practice nurses will be asked to reply by sending a photo of themselves and a copy of their e-signature. Individual practice posters will then be created by PHE and printed in A3 format by an external supplier (Williams Lea). Posters will also be laminated so that they are able to be cleaned (to ensure they meet health and safety regulations).

The commitment poster will contain a statement that:

Dear Patient,

We want to give you some information about antibiotics and caring for your health.

**Antibiotics** help us to fight serious infections caused by bacteria such as **meningitis, kidney infections and MRSA**.

But antibiotics can also cause side effects like skin rashes, diarrhoea, stomach pain, thrush or vomiting. If your symptoms are from a virus not from bacteria, like most colds, coughs, flu and some mild infections an antibiotic won’t help you get better and you could still get these side effects.

Taking antibiotics for illnesses which will not benefit from them encourages bacteria to become resistant to the treatment causing drug-resistant infections. This means that **antibiotics are losing their effectiveness**.

If you take antibiotics unnecessarily this could limit the treatment options available should you have more serious bacterial infections in the future. **Antibiotics might not work when you and your family really need them**.

**How can you help? Go to your pharmacist for advice if you have symptoms of the flu, a cold, cough, sinusitis, sore throat or a mild water, skin, eye or ear infections.** The pharmacist will give you advice on how to care for your infection and tell you whether you should see your GP.

As doctors we will always treat your illness in the best way possible. We will avoid prescribing antibiotics when they could do more harm than good.

**<*Insert name of practice*> are committed to combating drug resistant infections to protect your health and well-being. We will only prescribe antibiotics when it is the appropriate and necessary treatment. We do not prescribe antibiotics for colds and other self-limiting infections.**

**ELECTRONIC SIGNATURE AND PHOTOGRAPHS**

**PLEDGE TO CARE for patients & antibiotics**

Antibiotic prescribing – proceed with caution!

Poor prescribing puts patients at increased risk of drug resistant infections.

***Prescribe accordingly to guidelines***

***Leave self-limiting infections to resolve***

***Educate others***

***Don’t respond to inappropriate requests***

***Get a culture if appropriate***

***Effective prescribing reduces risk – for patients – for you community***

**Interventions 1 and 2 combined**

The third arm of the trial will be delivering both interventions 1 and 2 as described above within the same practice.

**Control**

Usual care in the usual GP practice setting.

**Intervention fidelity**

The intervention fidelity will be assessed through telephone calls by the project team to the GP surgeries randomised to the call waiting message intervention and through spot visits by local PHE centre staff and/or CCG prescribing leads to a randomly selected sample of commitment poster intervention practices.

**1. Main trial:**

**Outcome measures**

There are no data collection requirements for GP surgeries. The study will use:

1. EPACT data on filled antibiotic prescriptions per 1000 of the population for a baseline period of 3 months and the 4 month intervention period, and for the same intervention period the previous year adjusted for STARPU and practice list size.
2. Secondary outcomes will include percentage of all filled antibiotic prescriptions that are for broad-spectrum antibiotics

**Additional data to inform the analysis will include**

3. Information on other AMR initiatives in place before or during the trial period

**Data collection**

Data on the prescribing rates in these practices is obtained from HSCIC’s records on practice level presentation data, and practice level patient loads, which are used to produce figures for the total number of antibiotics prescribed in a given month per 1000 head of population.

**Sample size**

Data on the prescribing rates in GP practices has been obtained from HSCIC’s records. This includes practice level presentation data, and practice level patient loads, which are used to produce figures for the total number of antibiotics prescribed in a given month per 1000 head of population weighted for STARPU. We make use of the figures for March to May 2015 for these analyses. We excluded practices above the 95th percentile for prescribing rates on advice from public health and AMR professionals to prevent inappropriate involvement with practices subject to measurement error or who were systematically different from the main population (such as including out-of-hours services). After excluding outliers, practices have a mean prescribing rate per 1000 head of population of 79.54, and a standard deviation of 20.02. As we intend to use a fixed effects regression model accounting for time trends with seasonal effects, and making use of the previous periods data for each practice we control for these to estimate our minimum detectable effect size. Using significance level of 0.05 and 80% power, for a three armed trial, a sample of 175 practices would give us a minimum detectable effect size of 3.09 doses per 1000 population. This amounts to a 3.9% reduction in prescribing which we believe is an achievable impact for this intervention.

We considered a range of options as seen in the table below:

|  | Table 4: effect sizes for fixed effect model with trend, detectable decreases in doses (80% power) | | | | | |
| --- | --- | --- | --- | --- | --- | --- |
| Sample Size | Detectable effect 2 arms | % Change | Detectable effect 3 arms | % Change | Detectable effect 4 arms | % Change |
| 36 | 5.74 | 7.18% | 7.11 | 8.9% | 8.35 | 10.5% |
| 72 | 4.01 | 5.06% | 4.91 | 6.2% | 5.71 | 7.2% |
| 108 | 3.24 | 4.07% | 3.98 | 5.0% | 4.62 | 5.8% |
| 144 | 2.79 | 3.50% | 3.43 | 4.3% | 3.98 | 5.0% |
| 150 | 2.74 | 3.45% | 3.33 | 4.2% | 3.92 | 4.9% |
| 175 | 2.48 | 3.13% | 3.09 | 3.9% | 3.61 | 4.5% |
| 200 |  |  | 2.9 | 3.6% | 3.37 | 4.2% |
| 225 |  |  | 2.73 | 3.4% | 3.2 | 4.0% |
| 250 |  |  | 2.59 | 3.3% | 3.01 | 3.8% |

**Analysis**

We will use a fixed effects regression model, with time trends accounting for seasonal effects, to estimate the effect of treatment status on prescribing. Analysis will control for baseline prescribing rates, CCG, practice size and practice population size.  Standard errors in analysis will take account of the possibility of clustering behaviour within CCGs.

We estimate an empirical specification using ordinary least squares as follows:

Dip=α +β1 Tip +β2 Ii +β3 Pp +Uip

Where D is the number of doses prescribed per 1000 population, for GP practice i, in time p

α is a constant, capturing mean level of prescribing amongst practices in the omitted category

T is a vector of binary variables indicator treatment status,

I is a vector of practice specific fixed effects,

and P is a linear time trend of seasonal effects,

U is a standard error, assumed to be independent and individually distributed

**2. Qualitative study and short survey**

**Data Collection**

Semi-structured interviews will be carried out with general practitioners (GPs) by telephone. Interviews will follow a guide informed by existing research on GP antibiotic prescribing and the theoretical domains framework (TDF).^11^ Interview questions will ask about views on antibiotic prescribing, experiences of following the intervention(s) and any influence of the interventions on practice. Hypothesised mechanisms of action of the intervention will be assessed using constructs from the TDF. The semi-structured format will ensure that all participants are asked the central questions but will allow participants the opportunity to talk about additional issues of importance to them.^12^ Interview guides will be piloted with GPs from practices who have not participated in the trial to ensure questions are understandable and relevant to the target group. Interviews will last between 30 and 60 minutes.

A short questionnaire will be sent to practice managers of all practices who took part in the trial. Questionnaire items will ask about the ease of implementing interventions and any support given to practice staff to follow the interventions.

**Sample**

GPs will be invited to take part in the study by post, phone and/or email. Participants will be purposively sampled in order to obtain a maximum variation sample reflecting trial arm allocation, practice prescribing rate, practice list size and location in England.^13^ Approximately 30 GPs will be interviewed depending on data saturation (when no new themes emerge from interviews).^12^

Practice managers will be sent a link to an electronic version of the survey by email and will be invited to complete it online. Papers copies will be available on request. Practice managers will be contacted up to three times to complete the survey. The questionnaire will be delivered using Qualtrics survey software.

**Analysis**

Interviews will be audio recorded, professionally transcribed verbatim and managed using NVivo software. The data will initially be analysed using inductive thematic analysis by one member of the research team.^14^ Data will be coded, line by line, following a constant comparison technique.^15^ Codes will be examined for similarities and differences to categorise them into initial themes. Themes will be examined by other members of the research team and compared with the original data. Themes will also be considered in reference to the TDF to identify areas of overlap. Any disagreements will be resolved by consensus.Survey data will be analysed using descriptive statistics.

**Quality Assurance**

A stringent review process will be followed to ensure appropriateness of the two interventions:

Step 1: Internal review amongst PHE advisors; Elizabeth Beech, Susan Hopkins, Diane Ashiru-Oredope and Cliodna McNulty

Step 2: External review amongst CCG leads/ informal GP advisor panel

**Roles and responsibilities**

Anna Sallis, Project Lead, PHE ([anna.sallis@phe.gov.uk](mailto:anna.sallis@phe.gov.uk))

Tim Chadborn, Project Supervisor, PHE (tim.chadborn@phe.gov.uk)

Michael Sanders, Statistics, Behavioural Insights Team

**Confidentiality and ethical approval**

We will comply with data protection and confidentiality regulations.

NHS Proportionate Review and local NHS R&D approval will be sought.

**Funding**

The project will be funded by the PHE Behavioural Insights Team. The qualitative work will be funded by the PHE Behavioural Insights Team and the National Institute for Health Research Health Protection Research Unit (NIHR HPRU) in Healthcare Associated Infections and Antimicrobial Resistance at the University of Oxford.

**References**

1. Public Health England (2014). From Evidence into Action: Opportunities to Protect and Improve the Nation’s Health. Accessed on April 13, 2015 from https://www.gov.uk/government/uploads/system/uploads/attachment_data/file/366852/PHE_Priorities.pdf.
2. Department of Health (2013). UK Five Year Antimicrobial Resistance Strategy 2013 to 2018. Accessed on April 13, 2015 from https://www.gov.uk/government/uploads/system/uploads/attachment_data/file/244058/20130902_UK_5_year_AMR_strategy.pdf.
3. ESPAUR Report (2014). Accessed on April 13, 2015 from https://www.gov.uk/government/uploads/system/uploads/attachment_data/file/362374/ESPAUR_Report_2014__3_.pdf.
4. Public Health England (2015). Behaviour change and antibiotic prescribing in healthcare settings. Literature review and behavioural analysis. Accessed on April 13, 2015 from <https://www.gov.uk/government/uploads/system/uploads/attachment_data/file/405031/Behaviour_Change_for_Antibiotic_Prescribing_-_FINAL.pdf>.
5. Kumar S., Little P., Britten N. (2003). Why do general practitioners prescribe antibiotics for sore throat? Grounded theory interview study. *British Medical Journal*, 326: 138–8.
6. Changed from top 50% of prescribers only.
7. Solutions Research (2015) Antibiotics Proposition Research for PHE.
8. National Institute for Health and Care Excellence (2008). Respiratory tract infections – antibiotic prescribing: Prescribing of antibiotics for self-limiting respiratory tract infections in adults and children in primary care. NICE Guidelines CG69. Accessed on April 14, 2015 from <http://www.nice.org.uk/guidance/cg69/chapter/1-guidance>.
9. NHS Choices (2015). Respiratory Tract Infections. Accessed on 14 April, 2015 from <http://www.nhs.uk/conditions/Respiratory-tract-infection/Pages/Introduction.aspx>.
10. Royal College of General Practitioners (2015). Treating your infection patient leaflet. Accessed on 21 April, 2015 from <http://www.rcgp.org.uk/clinical-and-research/target-antibiotics-toolkit/patient-information-leaflets.aspx>.
11. Meeker, D., Knight, T.K., Friedberg, M.W., Linder, J.A., Goldstein, N.J., Fox, C.R., Rothfeld, A., Diaz, G. & Doctor, J.N. (2014). Nudging Guideline-Concordant Antibiotic Prescribing: A Randomized Controlled Trial. *JAMA Intern Med.* doi:10.1001/jamainternmed.2013.14191.

    ^11^ Cane J, O’Connor D, Michie S. (2012). Validation of the theoretical domains framework for use in behaviour change and implementation research. Implementation Science, 7, 37.

    ^12^ Hamberg, K., Johansson, E., Lindgren, G., Westman, G. (1994). Scientific rigour in qualitative research - examples from a study of women’s health in family practice. Fam Pract, 11 (2), 176-181.

    ^13^ Patton M. Qualitative Evaluation and Research Methods. Beverly Hills, CA: Sage; 1990.

    ^14^ Braun, V., Clarke, V. (2006). Using thematic analysis in psychology. Qual Res Psychol, 3 (2),77-101.

    ^15^ Glaser BG, Strauss AL. The Discovery of Grounded Theory: Strategies for Qualitative Research. New York, NY: Aldine De Gruyter; 1967.
